# Low Prevalence of *ETV6::RUNX1* Fusion Gene in a Hispanic Population

**DOI:** 10.3389/fped.2022.837656

**Published:** 2022-05-24

**Authors:** Minerva Mata-Rocha, Angelica Rangel-López, Elva Jimenez-Hernandez, Juan Carlos Nuñez-Enríquez, Blanca Angélica Morales-Castillo, Norberto Sánchez-Escobar, Omar Alejandro Sepúlveda-Robles, Juan Carlos Bravata-Alcántara, Alan Steve Nájera-Cortés, María Luisa Pérez-Saldivar, Janet Flores-Lujano, David Aldebarán Duarte-Rodríguez, Norma Angélica Oviedo de Anda, Maria de los Angeles Romero Tlalolini, Carmen Alaez Verson, Jorge Alfonso Martín-Trejo, Jose Esteban Muñoz Medina, Cesar Raul Gonzalez-Bonilla, Maria de los Angeles Hernandez Cueto, VC. Bekker-Méndez, Silvia Jiménez-Morales, Aurora Medina-Sansón, Raquel Amador-Sánchez, José Gabriel Peñaloza-González, José Refugio Torres-Nava, Rosa Martha Espinosa-Elizondo, Beatriz Cortés-Herrera, Luz Victoria Flores-Villegas, Laura Elizabeth Merino-Pasaye, Maria de Lourdes Gutierrez-Rivera, Martha Margarita Velazquez-Aviña, Jessica Denisse Santillan-Juarez, Alma Gurrola-Silva, Gabriela Alicia Hernández Echáurregui, Alfredo Hidalgo-Miranda, José Arellano Galindo, Haydeé Rosas-Vargas, Juan Manuel Mejía-Aranguré

**Affiliations:** ^1^CONACyT-Unidad de Investigacion Medica en Genetica Humana, Hospital de Pediatria, Centro Medico Nacional Siglo XXI, IMSS, Mexico City, Mexico; ^2^Coordinacion de Investigacion en Salud, Unidad Habilitada de Apoyo al Predictamen, Centro Medico Siglo XXI, IMSS, Mexico City, Mexico; ^3^Servicio de Hematologia Pediatrica, Hospital General “Gaudencio González Garza”, Centro Medico Nacional (CMN) “La Raza”, IMSS, Mexico City, Mexico; ^4^Unidad de Investigacion Medica en Epidemiologia Clinica, Hospital de Pediatria, Centro Medico Nacional Siglo XXI, IMSS, Mexico City, Mexico; ^5^Unidad de Investigacion Medica en Genética Humana, Hospital de Pediatria, Centro Medico Nacional Siglo XXI, IMSS, Mexico City, Mexico; ^6^Facultad de Medicina y Cirugía, UABJO, Oaxaca, Mexico; ^7^Laboratorio de Genética y Diagnóstico Molecular, Hospital Juárez de México, Secretaría de Salud (SSa), Mexico City, Mexico; ^8^UIM en Inmunología e Infectología, Centro Médico Nacional “La Raza”, IMSS, Mexico City, Mexico; ^9^Laboratorio de Diagnóstico Genómico, Instituto Nacional de Medicina Genómica (INMEGEN), Mexico City, Mexico; ^10^Servicio de Hematologia, UMAE Hospital de Pediatria, Centro Medico Nacional Siglo XXI, IMSS, Mexico City, Mexico; ^11^Laboratorio Central de Epidemiología, Centro Médico Nacional “La Raza”, Instituto Mexicano del Seguro Social, Mexico City, Mexico; ^12^Profesor de Asignatura de la Facultad de Medicina, UNAM, Mexico City, Mexico; ^13^Centro Médico Nacional La Raza, División de Laboratorios de Vigilancia e Investigación Epidemiológica, Instituto Mexicano del Seguro Social, Mexico City, Mexico; ^14^Laboratorio de Genomica del Cancer, Instituto Nacional de Medicina Genómica, Mexico City, Mexico; ^15^Servicio de Oncología, Hospital Infantil de Mexico Federico Gómez, Secretaria de Salud, Mexico City, Mexico; ^16^Servicio de Hematologia Pediatrica, Hospital General Regional “Carlos McGregor Sanchez Navarro”, IMSS, Mexico City, Mexico; ^17^Servicio de Onco-Pediatria, Hospital Juarez de Mexico, Secretaria de Salud, Mexico City, Mexico; ^18^Servicio de Oncología, Hospital Pediatrico de Moctezuma, Secretaria de Salud de la Ciudad de Mexico (SSCDMX), Mexico City, Mexico; ^19^Servicio de Hematologia Pediatrica, Hospital General de Mexico, Secretaria de Salud, Mexico City, Mexico; ^20^Servicio de Hematologia Pediatrica, Centro Medico Nacional “20 de Noviembre”, ISSSTE, Mexico City, Mexico; ^21^Oncología, UMAE Hospital de Pediatria, Centro Medico Nacional Siglo XXI, IMSS, Mexico City, Mexico; ^22^Servicio de Hemato-Oncologia Pediatrica, Hospital Regional 1° de Octubre, ISSSTE, Mexico City, Mexico; ^23^Servicio de Pediatría, Hospital Regional Tipo B de Alta Especialidad Bicentenario de la Independencia, Instituto de Seguridad Social al Servicio de los Trabajadores del Estado, Mexico City, Mexico; ^24^Unidad de Investigación en Enfermedades Infecciosas, Laboratorio de Virologia Clínica y Experimental, Hospital Infantil de Mexico Federico Gómez, Secretaria de Salud, Mexico City, Mexico; ^25^Facultad de Medicina, Universidad Nacional Autónoma de México, Mexico City, Mexico

**Keywords:** fusion gene, acute lymphoblastic leukemia, prognosis, RT-qPCR, molecular biomarkers

## Abstract

*ETV6::RUNX1* is a genetic rearrangement of good prognosis in children with acute lymphoblastic leukemia (ALL). In Mexico, its prevalence is low in comparison with Caucasian populations. We developed a novel TaqMan one-step RT-qPCR approach to assess the prevalence of four genetic rearrangements in a cohort of Hispanic children with ALL from Mexico City. The prevalence of common fusion gene transcripts was as follows: *TCF3::PBX1* 7.7%; *BCR::ABL1p*^190^ 3.3%; and *KMT2A::AFF1* 2.8%, and *ETV6::RUNX1*was observed with low prevalence (10.5%) in comparison to that reported for developed countries. This is consistent with previous findings on Mexican children with ALL and similar to those reported on children from Hispanic populations. The confirmation of a low prevalence of *ETV6::RUNX1* in children of a Hispanic origin represents an advancement in the description of genetic factors of ALL in these populations.

## Introduction

B-lineage acute lymphoblastic leukemia (B-ALL) is a malignant transformation of B-lymphoid cell precursors characterized by uncontrolled proliferation and accumulation of leukemic blasts in bone marrow (1). In recent times, the mortality of B-ALL has increased considerably in Latin America. B-ALL is the second leading cause of death in the group aged between 5 and 14 years (2), with an estimated incidence of 49.5 cases per million in Mexico City. This incidence is one of the highest reported in North America and is similar to Hispanics living in the United States, with a predicted increase in mortality by 2030 (3–5). Over the last few years, the survival of children with ALL has not improved in Mexico; according to the CONCORD-3 study, the five-year survival rate for Mexican children with ALL was <60%, while in developed countries rates reached 90% 5 years after diagnosis (6). This low survival of Mexican children with ALL is mainly related to a high percentage of relapses (26.2%), infections (14.2%), chemotherapy-related toxicity during the first year of treatment (7.1%), and a high frequency of mortality during the induction-to-remission phase (7%), among others (7, 8). Additionally, a study on medical care in childhood ALL suggests that improvements in quality of care are required, especially in individuals living in areas with limited access to a specialized healthcare system to avoid delay in treatment by oncology-hematology specialists, enable timely treatment, and avoid abandonment (9).

The presence of translocations in cancer reflects the genomic instability of cells and may characterize specific pathways involved in malignancy; however, mechanisms that cause these translocations are poorly understood. Translocations, often associated with hematological malignancies, have been postulated as one of the main causes of oncogenic transformations associated with leukemogenesis during fetal development (10, 11). However, they are not sufficient by themselves to trigger the leukemic process; additional cooperating mutations are usually required (12). Specific translocations have been associated with an increase in the risk of developing B-ALL, and these may result in the deregulation of one of the genes involved (overexpression of proto-oncogenes or inhibition of tumor suppressor gene) or in the expression of oncogenic proteins such as BCR-ABL1 (12, 13). The study on translocations has allowed for the characterization of four common subtypes in children with B-ALL, *KMT2A::AFF1, ETV6::RUNX1, BCR::ABL1p*^190^, and *TCF3::PBX1*, which alter gene expression and can disturb signaling pathways. The improved detection of fusion genes with prognostic significance may aid in risk stratification and identification of patients who need more aggressive therapy and those for whom less intense therapy is warranted in order to reduce toxicity and relapse risks (14, 15). Fusion gene detection has contributed to improved survival rates in children from other populations, mainly in developed countries (16). In relation to the detection of fusion genes in Mexico, diverse studies (9, 17) have reported low prevalence of favorable *ETV6::RUNX1* rearrangements (18–23). Interestingly, the prevalence of *ETV6::RUNX1* is also significantly lower in Hispanics than in non-Hispanic whites (24, 25). These studies reported that the low prevalence of *ETV6::RUNX1* may be the result of important ethnic and geographic differences related to the percentage of Native American and European genetic background in Mexicans (21). In Mexico, a FISH analysis revealed a low frequency of *ETV6::RU*N *X1* (8.5%); and abnormalities as extra *ETV6* and *RUNX1* copies as well as structural changes in *ETV6* in 44% of patients evidenced the different alterations that can occur in these genes in children with ALL (22, 23). In addition to the low prevalence of *ETV6::RUNX1* (6.9–14.9%) in México, in comparison to those reported in Caucasian populations (25%), the high prevalence of *TCF3::PBX1* (11.5%) has also been detected, which supports the existence of ethnic differences in the frequency of molecular markers of ALL (19). However, these studies in Mexico have small samples sizes that were analyzed by low-sensitivity/specificity techniques. In this study, we sought to reduce two possible sources of error from previous studies that could be causing a low frequency ofentification of genetic rearrangements. For our study, participation was improved to reduce possible selection biases, including more than 80% of children diagnosed with ALL in Mexico City from 2018 to 2019. We also optimized the detection of *ETV6::RUNX1, KMT2A::AFF1, BCR::ABL1p*^190^, and *TCF3::PBX1* fusions by TaqMan one-step RT-qPCR, which is a method with higher sensitivity and specificity than previously used techniques.

## Methodology

### Development and Validation of the Method

We developed and validated a TaqMan one-step RT-qPCR method to optimize the detection of *ETV6::RUNX1, KMT2A::AFF1, BCR::ABL1p*^190^, and *TCF3::PBX1* fusions. An overview of the development and validation of the method described here is presented in Supplementary Figure 1. To design primers and probes, we used the Primer3Plus software program; the position and sequences of the primers and probes are shown in Supplementary Figure 2 and Supplementary Table 1. To validate the method, we used total RNAs derived from Reh (ATCC® CRL-8286™, RRID: CVCL_1650), SUP-B15 (ATCC® CRL-1929, RRID: CVCL_0103), and RS4;11 (ATCC® CRL-1873, RRID: CVCL_0093) leukemia cell lines. For repeatability evaluation, the detection of each transcript was independently tested four times on 5 days by the same operator, while for reproducibility each fusion was analyzed four times by three different operators on 3 different days for all fusion genes and the internal control of glyceraldehyde-3′-phosphate dehydrogenase (GAPDH). Sensitivity was compared with QuanDxLeukemia Fusion Genes (Q30) Screening Kit (a multiplex system for simultaneous detection of 30 fusion genes in patients with leukemia) in a separate laboratory (Hospital Juarez de Mexico). Forty-five clinical samples as well as twenty-five fusion gene-positive samples outside the cohort were blindly analyzed with both the QuanDXleukemia (Q30) kit and the TaqMan one-step RT-qPCR method (Supplementary Tables 3, 4). Plasmids containing the *GAPHD, ETV6::RUNX1, KMT2A::AFF1, BCR::ABL1p190*, and *TCF3::PBX1* transcript sequences were generated by cloning amplified PCR products into a pJET1.2 vector using CloneJET™ PCR Cloning Kit (Thermo Fisher Scientific™). Then, the plasmids were transformed and propagated in *E. coli* JM109 and extracted using the GeneJET Plasmid Miniprep Kit (Thermo Fisher Scientific™) and sequenced using an ABI3500xL genetic analyzer (Applied Biosystems, Waltham, MA, United States). Plasmid DNA concentration was calculated using a Qubit dsDNA BR assay (Life Technologies Co., Carlsbad, CA, United States) to improve the accuracy of quantification. We converted DNA plasmid size to copy number by multiplying the number of base pairs of each plasmid by the average molecular mass of one base pair (~660 g/mol) to get the approximate mass and then divided it by Avogadro's constant: NA = 6.022 × 10^23^ molecules. The plasmids were serially diluted, and linear regression was performed between the cycle threshold value (Ct-value) and the log10 of the copy number. Amplification efficiency for each fusion reaction was calculated using the equation: *E* = 10(−1/slope) and converted to *E*% by (*E* – 1) × 100.

### Patients

This study was conducted from January 2018 to December 2019 with B-ALL cases. From a total of 279 cases from eight public hospitals in Mexico City, 247 available bone marrow samples were selected for one-step RT-qPCR analysis. The diagnosis of B-ALL was based on the morphology of leukemic cells and immunophenotyping. Table 1 contains relevant information about patients. The patients included had at least 18 months of follow-up. This study followed accepted principles of ethical and professional conduct according to approval by the National Ethics and Scientific Committee of IMSS (R-2015-785-121). Treatment protocols were: 1) St. Jude Total XIIIB; 2) modified BFM-95; 3) Dana-Farber Cancer Institute 00-01. Written informed consent was obtained from the children's parents to participate in the study.

**Table 1:** Clinical features of patients with acute lymphoblastic leukemia (ALL) by molecular subtype.

	***ETV6::RUNX1***	***TCF3::PBX1***	***BCR::ABL1 p^**190**^***	***KMT2A::AFF1***	**Non-detected**	**Included patients**	**Not included patients**	*P-*value^**^
Totalnumbers	26	19	8	7	187	247	32	
**Clinicalsubgroups**	*n* (%c) (%r)	*n* (%c) (%r)	*n* (%c) (%r)	*n* (%c) (%r)	*n* (%c) (%r)	*n* (%c) (%r)	*n* (%)	
**Sex**								
Male	18 (69.2) (13.5)	9 (47.4) (6.8)	4 (50.0) (3.0)	4 (57.1) (3.0)	98 (52.4) (73.7)	133 (53.8) (86.4)	21 (65.6) (13.6)	0.2
Female	8 (30.8) (7.0)	10 (52.6) (8.8)	4 (50.0)(3.5)	3 (42.9) (2.6)	89 (47.6) (78.1)	114 (46.2) (91.2)	11 (34.4) (0.8)	
**Age subgroups (years)**								
<1	0 (0.0) (0.0)	0 (0.0) (0.0)	0 (0.0) (0.0)	0 (0.0) (0.0)	3 (1.6) (100)	3 (1.2) (75)	1 (3.1) (25)	0.39
1–5.99	15 (57.7) (17.4)	8 (42.1) (9.3)	1 (12.5) (1.2)	1 (14.3) (1.2)	61 (32.6) (70.9)	86 (34.8) (85.1)	15 (46.9) (14.9)	
6–9.9	6 (23.1) (10.0)	3 (15.8) (5.0)	2 (25.0) (3.3)	2 (28.6) (3.3)	47 (25.1) (78.3)	60 (24.3) (92.3)	5 (15.6) (7.7)	
10–14.9	2 (7.7) (2.8)	7 (36.8) (9.8)	4 (50.0) (5.6)	2 (28.6) (2.8)	56 (29.9) (78.9)	71 (28.7) (86.5)	11 (34.4) (13.5)	
≥15	3 (11.5) (11.1)	1 (5.3) (3.7)	1 (12.5) (3.7)	2 (28.6) (7.4)	20 (10.7) (74.0)	27 (10.9) (100)	0 (0.0) (0.0)	
**WBC count (cells/ul)**								
<20,000	24 (92.3) (10.2)	17 (89.5) (7.2)	7 (87.5) (3.0)	6 (85.7) (2.6)	181 (96.8) (77.0)	235 (95.1) (90.4)	25 (78.1) (9.6)	<0.001
≥20,000–50,000	2 (7.7) (33.3)	1 (5.3) (16.7)	0 (0.0) (0.0)	0 (0.0) (0.0)	3 (1.6) (50.0)	6 (2.4) (75)	2 (6.2) (25)	
≥50,000	0 (0.0) (0.0)	1 (5.3) (16.7)	1 (12.5) (16.7)	1 (14.3) (16.7)	3 (1.6) (50.0)	6 (2.4) (54.5)	5 (15.7) (45.5)	
^*^NCI-riskclassification								
Standard	16 (615) (12.4)	8 (42.1) (6.2)	1 (12.5) (0.8)	1 (14.3) (0.8)	103 (55.1) (79.8)	129 (52.2) (88.4)	17 (53.1) (11.6)	0.92
High	10 (38.5) (8.5)	11 (57.9) (9.3)	7 (87.5) (5.9)	6 (85.7) (5.1)	84 (44.9) (71.2)	118 (47.8) (88.7)	15 (46.9) (11.3)	
**Relapse**								
Yes	0 (0.0) (0.0)	1 (5.3) (8.3)	1 (12.5) (8.3)	3 (42.9) (25.0)	7 (3.7) (58.3)	12 (4.8) (80)	3 (9.4) (20)	0.28
No	26 (100.0) (11)	18 (94.7) (7.6)	7 (87.5) (3.0)	4 (57.1) (1.7)	180 (96.3) (76.7)	235 (95.2) (89)	29 (90.6) (11)	
**Death**								
Yes	1 (3.8) (6.7)	1 (5.3) (6.7)	2 (25.0) (13.3)	1 (14.3) (6.7)	10 (5.3) (66.6)	15 (6.0) (78.9)	4 (12.5) (21.1)	0.17
No	25 (96.2) (10.8)	18 (94.7) (7.7)	6 (75.0) (2.6)	6 (85.7) (2.6)	177 (94.7) (76.3)	232 (94.0) (89.2)	28 (87.5) (10.8)	

* *According to the National Cancer Institute Risk Classification, patients were classified as standard risk (NCI SR) [with age ranging from 1 to 9.99 years and an initial white blood cell (WBC) count <50 ×109/L], or as high risk (NCI HR) [age <1 or ≥ 10 years or initial WBC ≥50 ×109/L (27)*.

** *To compare clinical characteristics between analyzed and non-analyzed ALL patients, the chi-square test or Fisher's exact test was conducted when appropriate. n (%c) (%r): number of cases in percentage per column and per row obtained from each clinical subgroup, respectively*.

### RNA Isolation From Bone Marrow Samples

Total RNA was extracted using the Direct-zol MiniPrep (Zymo Research, United States) according to the manufacturer's protocol. The quality of RNA was examined by analysis of *GAPDH* transcript copy numbers determined by plasmid DNA standard curves, which should be >2.5 × 10^5^ copies/ul, as has been previously reported (26). Only samples with sufficient quality were analyzed.

### Reverse Transcription–Polymerase Chain Reaction for Fusion Gene Detection

After validation, fusion gene detectio*n* (*ETV6::RUNX1, TCF3::PBX1, BCR::ABL1p190, KMT2A::AFF1, GAPDH*) was carried out with 5 μl of 2× QuantiTect Probe RT-PCR Master Mix and HotStarTaq® DNA Polymerase using 0.5 μl QuantiTect RT Mix, 200 nM of each primer, and 500 nM of each TaqMan probe in a final volume of 50 μl. Thermal cycling conditions were as follows: 30 min at 50°C and 15 min at 95°C followed by 38 cycles of 94°C for 15 s and 60°C for 1 min.

### Statistical Analysis

Statistical analyses were performed using SPSS IBM (Statistical Package for the Social Sciences, Inc., Version 21, Chicago, IL, United States). The prevalence of the four most common gene rearrangements in pediatric ALL was determined. The Kaplan-Meier (KM) method was used to estimate disease-free survival (DFS) and overall survival (OS), and a log-rank test was conducted to compare the survival curves at the <0.05 level of significance. DFS was defined as the time from documented complete remissio*n* (CR) to relapse or death from any cause.

## Results

### Development and Validation of TaqMan One-Step RT-QPCR for Detection of *ETV6::RUNX1, KMT2A::AFF1, BCR::ABL1p^190^*, and *TCF3::PBX1* Fusion Genes

We developed and validated a one-step TaqMan RT-qPCR method for the detection of the fusion gene transcripts *ETV6::RUNX1, KMT2A::AFF1, BCR::ABL1p*^190^, and *TCF3::PBX1*. The use of a one-step assay reduces bench time and, thus, pipetting errors, as well as cross-contamination between RT and PCR steps. We can detect fusion genes using 50-100 ng of total RNA, with a detection sensitivity of ≥100 copies/μl of fusion transcripts. The standard curves using plasmid produced linear results, with high amplification efficiencies for the *GAPDH* (107%), *ETV6::RUNX1* (101%), *KMT2A::AFF1* (97%), *BCR::ABL1p*^190^ (98%), and *TCF3::PBX1* (106%) transcripts, indicating an optimal PCR yield in which the number of copies doubles in each cycle (Supplementary Figure 3). To assess inter-laboratory variation, we obtained good precision, since the coefficients of variatio*n* (CV) of reproducibility and repeatability were 2.1–3 and 2.6–3.8%, respectively (Supplementary Table 2). Assay specificity was evaluated by testing bone marrow RNA from patients without ALL but with other diseases such as pancytopenia, thrombocytopenia, hemophagocytic histiocytosis, or viral infections such as cytomegalovirus. We also use a mixture of different plasmids: *EP300-ZNF384, CREBBP-SRGAP2B, DNAH14-IKZF1, ETV6-SNUPN*, and *ETV6-NUFIP1* fusion genes. RNA and plasmids were negative for the common fusion genes (Ct value < 40). The validation was completed by independent testing of 45 and 25 fusion genes positive for the fusion gene samples in an external laboratory (Hospital Juarez). To this end, our collaborators detected fusion transcripts using the QuanDX kit, while in parallel we analyzed the same samples with the TaqMan one-step RT-qPCR method. The results for *ETV6::RUNX1, KMT2A::AFF1, BCR::ABL1p*^190^, and *TCF3::PBX1* fusion genes detection by the two methods showed a perfect correlation (Supplementary Tables 3, 4). From the 45 samples, only 7 were detected as positive for any of the transcripts by both methods (15.5%), and three were positive for *ETV6::RUNX1*, three were positive for *TCF3::PBX1*, and two were positive for *BCR::ABL1p*^190^ (Supplementary Table 3).

### Performance of TaqMan One-Step RT-qPCR on Clinical Samples to Determine the Prevalence and Prognostic Impact of Fusion Genes

**Figure 1:**
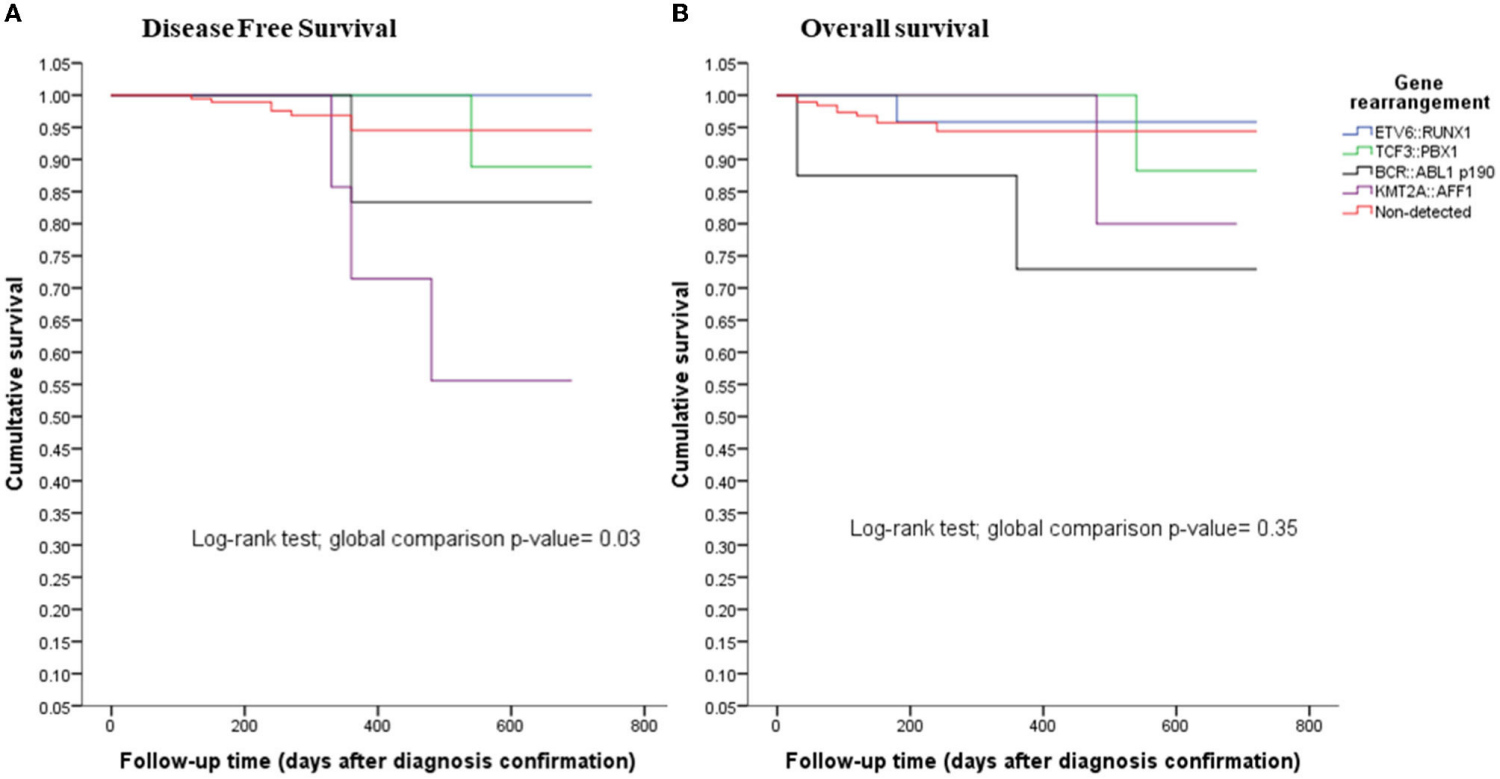
**(A)** disease-free survival and **(B)** overall survival rates for childhood with acute lymphoblastic leukemia (ALL) by detected fusion gene.

The Mexican Interinstitutional Group for theentification of the Causes of Childhood Leukemia (MIGICCL) collected 279 samples of BM from children with newly diagnosed ALL from 2018 to 2019. Of these, a total of 247 samples had enough RNA quantity (50–100 ng/ul) and good quality of RNA that was examined by *GAPDH* (Ct value < 20) (26). The TaqMan one-step RT-PCR was conducted (Table 1). We performed a comparison between the clinical characteristics of patients with ALL analyzed and non-analyzed, and the variables of both groups were not similar (***p*** > 0.05), so we consider that our cohort is not biased (Table 1). The children had a mean age of 8.6 years (range 0.2–17.7 years), of which 133 were male (53.8%). During the follow-up, 15 (6%) of the patients died and 12 (4.8%) relapsed. In total, 75.7% were negative for the **four** fusion genes analyzed, while only 24.3% (60/247) were positive. The prevalence of the fusion gene transcripts was as follows: *ETV6::RUNX1* (10.5%), *TCF3::PBX1* (7.7%), *KMT2A::AFF1* (2.8%), and *BCR*::*ABL1p*^190^ (3.3%) (Table 1). The Kaplan-Meier estimations for overall survival (OS) and disease-free survival (DFS) according to fusion gene transcripts are displayed in Figure 1. A different OS for each fusion gene was noted in the cohort, however, solid conclusions about the impact of gene fusion on the OS and DFS of Mexican children with ALL may require longer follow-up times. *ETV6::RUNX1* was the most common fusion gene transcript present in all age groups; however, the analysis of *ETV6::RUNX1* for age groups showed that this fusion was observed mainly in children 1–6 years old in 57.5% of the cases, who were predominantly male (73.3%). Of the patients with *ETV6::RUNX1*, 92.3% had a white blood cell count below 20,000, and 61.5% were classified as standard risk according to the NCI classification. In this study, almost all the patients with the *ETV6::RUNX1* fusion gene remained in first complete remission at the time of conclusion of this study; only one patient exhibited poor response and died (Table 1). The lowest prevalence of fusion gene transcript was *KMT2A::AFF1*, observed in 2.8% of patients aged 1–16 years at the time of diagnosis. Approximately 85.7% of them were classified as high-risk according to the NCI classification, three relapsed, and one died. On the other hand, the *TCF3::PBX1*fusion gene was present in 19 patients (7.7%) aged 1-16 years. Of these patients, 89.5% had a white blood cell count below 20,000 (cells/ul) at the time of diagnosis. Eleve*n* (57.9%) were classified as high risk, one relapsed, and one patient died. Finally, transcribed *BCR::ABL1p*^190^showed a positivity rate of approximately 3.6%, and most of the patients had complete remission. One relapse and two deaths occurred in this molecular subgroup.

## Discussion

In children with ALL who were attended at public hospitals in Mexico City, identification of common fusion genes is not routinely performed; however, it may be available through research projects such as this study. For this purpose, we used the TaqMan one-step RT-qPCR method toentify the prevalence of the *ETV6::RUNX1* (10.5%), *TCF3::PBX1* (7.7%), *KMT2A::AFF1* (2.8%), and *BCR::ABL1p*^190^ (3.6%) fusion genes and their association with early mortality in patients with B- ALL. The overall prevalence was 24.2%, which was relatively higher than in previous studies carried out by our research group (17.7%) (18), and 18.83% in a study performed in southern Mexico (20). Also, we detected a higher average age of 8.6 years (range.2–17.7 years) than that of Caucasian children with ALL (5.4) (28), similar to a recent study in Mexico, which is 9.3 (< 1–19, N = 154, 2020 B-ALL (20). This increase is the result of a higher percentage in the cohort of patients with ALL over 10 years of age (39.7%) (Table 1) related to the increase reported on ALL in the Latin American population (5).

In addition to analyzing the prevalence of major fusion genes for stratification of patients with ALL, we implemented improvements in the analysis technique to have greater sensitivity and reproducibility with respect to previously described studies. We were able to increase acceptance among patients with B- ALL to participate in the study, and we contrasted good and poor prognostic rearrangements with early mortality. This study confirmed the presence of a low prevalence of the *ETV6::RUNX1* fusion gene (10.5%) and was within the range of previous Mexican reports using conventional RT-PCR of 7.4% (18), 13.5% (19); fluorescence *i*n *situ* hybridization of 8.5% (22), 8.7% (23); RT- Multiplex PCR 6.9% (20), 14.9% (21). The low prevalence of *ETV6::RUNX1* is similar to other Hispanic populations such as 4.5% in Guatemalans and 14% in Hispanic residents of Northern California (24, 25). Therefore, the low prevalence of *ETV6::RUNX1* is probably due to the genetic background of Hispanic populations. In the same context, it has been observed that the incidence of ALL subtypes and genetic susceptibility to ALL varies according to geographic regions and ancestry of populations. Recently, it has been reported that in a population with a higher prevalence of Native American ancestry, the *ETV6::RUNX1* fusion gene is found with lower frequency, and this population was associated with poor prognosis (29). This directly correlates with the ancestry analysis that was performed in Mexican ALL patients using AIMs (Ancestry-informative marker, single nucleotide polymorphism of frequency differences between populations) which showed that they belong to the mestizo group, enriched mainly by a Native American (30). Also, it has been reported that genetic variants are associated with ALL in an ethnic-specific manner and may give rise to racial differences in ALL incidences. For example, *ARID5B* rs10821936 polymorphisms exhibited significant association with ALL susceptibility, were highly correlated with local Native American genetic ancestry, and were significantly overrepresented in the hyperdiploid subtype (31). In Mexico, we have reported that *ARID5B* and *NAT2* polymorphisms are associated with susceptibility to childhood ALL in a Mexican population (30). Therefore, these polymorphisms, together with the low prevalence of *ETV6::RUNX1*, contribute to deciphering the genetics of ALL in Mexican children, a population related to poor prognosis.

However, it also raises concerns on whether the low prevalence of *ETV6::RUNX1* is due to misdiagnosis. In this sense, previous studies on prevalence of common fusion genes in Mexico have been carried out with conventional methods with lower sensitivity as RT-PCR, FISH, karyotype. In this study, RT-qPCR was used, a sensitive method for detection of fusion genes in tumor samples that conventional methodologies detecting even a single molecule in lower amounts of complex samples as bone barrow, which supports the low prevalence of *ETV6::RUNX1* obtained (32, 33).

Regarding other translocations that were analyzed, the prevalence that has been reported for *TCF3::PBX1*-positive ALL varies between 3 and 5% in childhood, while we showed a major prevalence of 7.5%, distributed across all age groups. In *TCF3::PBX1-*positive ALL, the prognosis has improved remarkably in the last years under the current treatment in developed countries. The situation might be different in developing countries (34–36). In this study *TCF3::PBX1-*positive ALL showed better DFS than *KMT2A::AFF1-* and *BCR::ABL1p*^190−^positive ALL, suggesting that the current treatment protocols used in Mexico show improvements in early mortality. Conversely, patients with *KMT2A::AFF1* showed low DFS rates as detected in children between 4 and 16 years of age. This correlates with the analysis of the KMT2A recombinome in acute leukemia and its distribution in clinical subgroups where AFF1 is the most frequent KMT2A rearrangement in ALL present in infant, pediatric, and adult patients (37). Of note, we do not have infant patients in the analysis of this study. Finally, *BCR::ABL1* translocation is associated with poor prognosis; this encodes a tyrosine kinase, which is considered a pathogenetic driver that can be therapeutically targeted (38), and so, fortunately, treatment with tyrosine kinase (TK) inhibitors has improved the overall survival of patients with this rearrangement (39). In this study, *BCR::ABL1p*^190^ translocation wasentified in 3.6% of the patients and showed low DFS than the other fusion genes, similar to other studies (40). The follow-up time is short at this moment; however, the survival analysis is evidence of the concordance between the genetic rearrangement and the prognosis of the disease. However, we acknowledge that further data are required to more firmly establish the prognostic impact of the detected fusions.

## Conclusion

The detection of the most common fusion transcripts in the Mexican population and, in particular, the finding of the low prevalence of ETV6::RUNX1 represent an advancement in the description of the genetic factors in the Mexican childhood population that are associated with the stratification of the molecular subtypes of ALL that is fundamental to implementing better treatments in a personalized manner to increase the survival rate.

## Data Availability Statement

The original contributions presented in the study are included in the article/Supplementary Material, further inquiries can be directed to the corresponding author/s.

## Ethics Statement

The studies involving human participants were reviewed and approved by Comisión de Etica y Científica Instituto Mexicano del Seguro Social. Written informed consent to participate in this study was provided by the participants' legal guardian/next of kin.

## Author Contributions

JM-A, JN-E, EJ-H, and HR-V participated in the main research idea, work supervision, data analysis, and manuscript preparation and revision. MM-R and AR-L participated in the experimental design, shared research ideas, carried out the experiments, and wrote the first draft of the manuscript. BM-C, NS-E, JB-A, AN-C, and MH carried out and analyzed the experiments and the manuscript. JM, CG-B, and OS-R participated in the experimental idea and in writing the final version of the manuscript. MP-S, JF-L, DD-R, NO, CA, MR, JM-T, AM-S, RA-S, JP-G, JT-N, RE-E, BC-H, LF-V, LM-P, MV-A, JS-J, AG-S, GH, VB-M, SJ-M, AH-M, and JA participated in resources, data collection, and literature search. All the authors have read and agreed to the published version of the manuscript.

## Funding

This study was supported by the Consejo Nacional de Ciencia y Tecnología (CONACyT) under grants [PDCPN2013-01-215726, FIS/IMSS/PROT/1364, SALUD 2015-1-262190, FIS/IMSS/PROT/1533, CB-2015-1-258042, FIS/IMSS/PROT/1548, FONCICYT/37/2018, FIS/IMSS/PROT/1782, FORDECYT-PRONACES/303019/2019, and FORDECYT-PRONACES/377883/2020], the Instituto Mexicano del Seguro Social under grants [FIS/IMSS/PROT/PRIO/14/031, FIS/IMSS/PROT/PRIO/15/048, FIS/IMSS/PROT/PRIO/18/080, FIS/IMSS/PROT/PRIO/19/088], and publication was paid by Instituto Nacional de Medicina Genómica.

## Conflict of Interest

The authors declare that the research was conducted in the absence of any commercial or financial relationships that could be construed as a potential conflict of interest.

## Publisher's Note

All claims expressed in this article are solely those of the authors and do not necessarily represent those of their affiliated organizations, or those of the publisher, the editors and the reviewers. Any product that may be evaluated in this article, or claim that may be made by its manufacturer, is not guaranteed or endorsed by the publisher.
