# Supplementary figures and images for: Low Prevalence of ETV6::RUNX1 Fusion Gene in a Hispanic Population
Source: Front Pediatr. 2022 May 24;10:837656. doi: 10.3389/fped.2022.837656 (PMC9171364; doi:10.3389/fped.2022.837656)

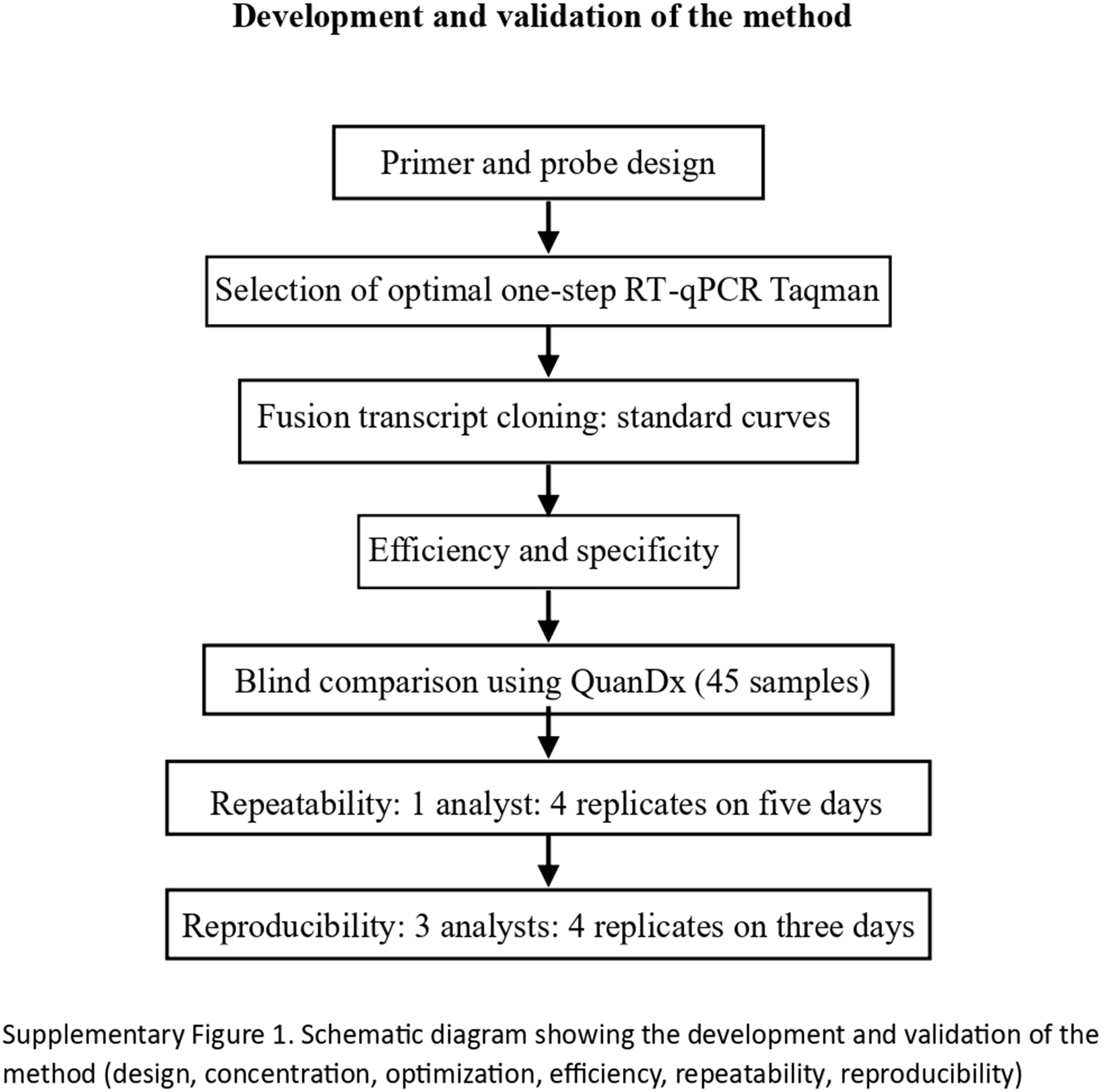

Supplement: Supplementary file 5 [file Image_1.JPEG]

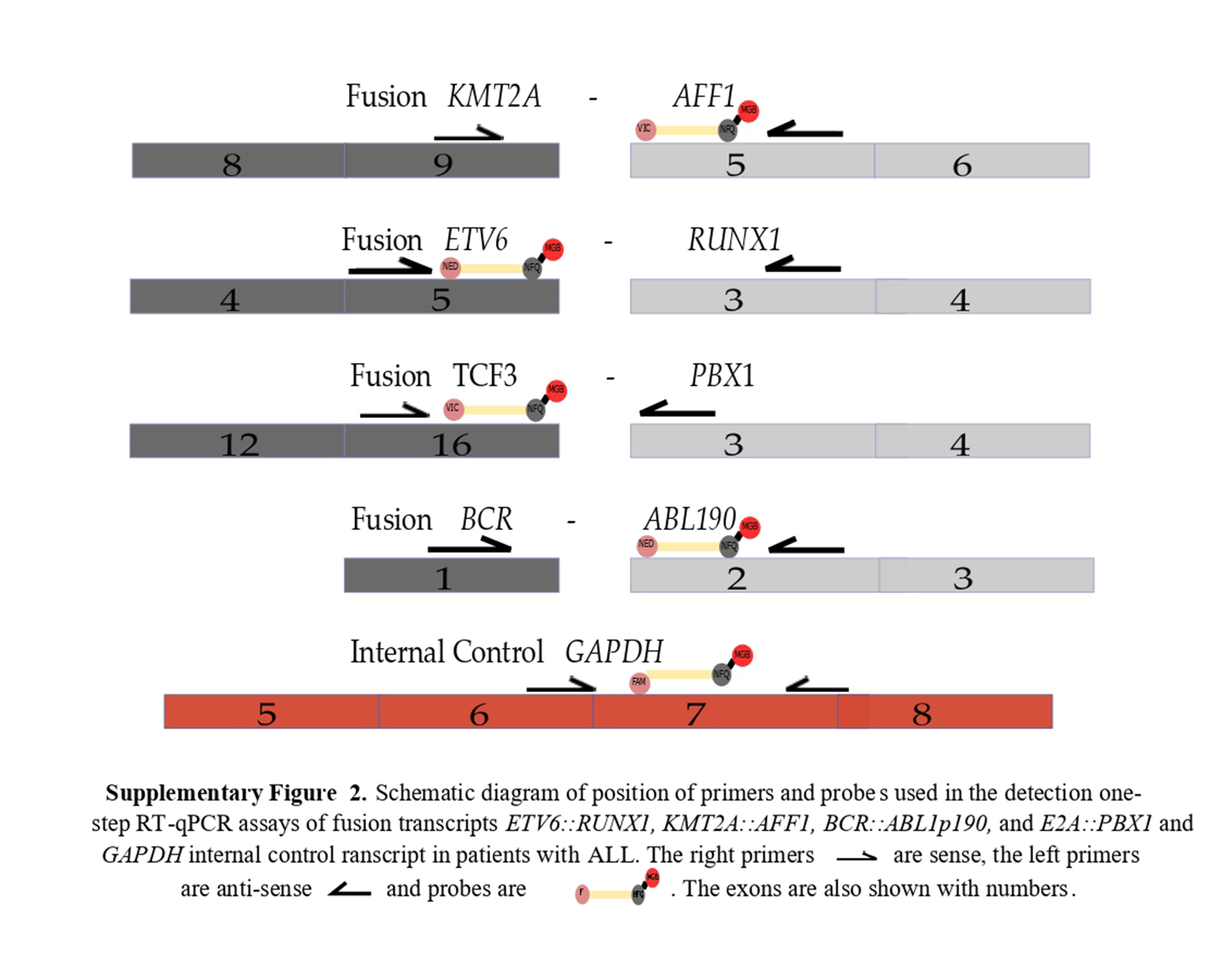

Supplement: Supplementary file 6 [file Image_2.jpeg]

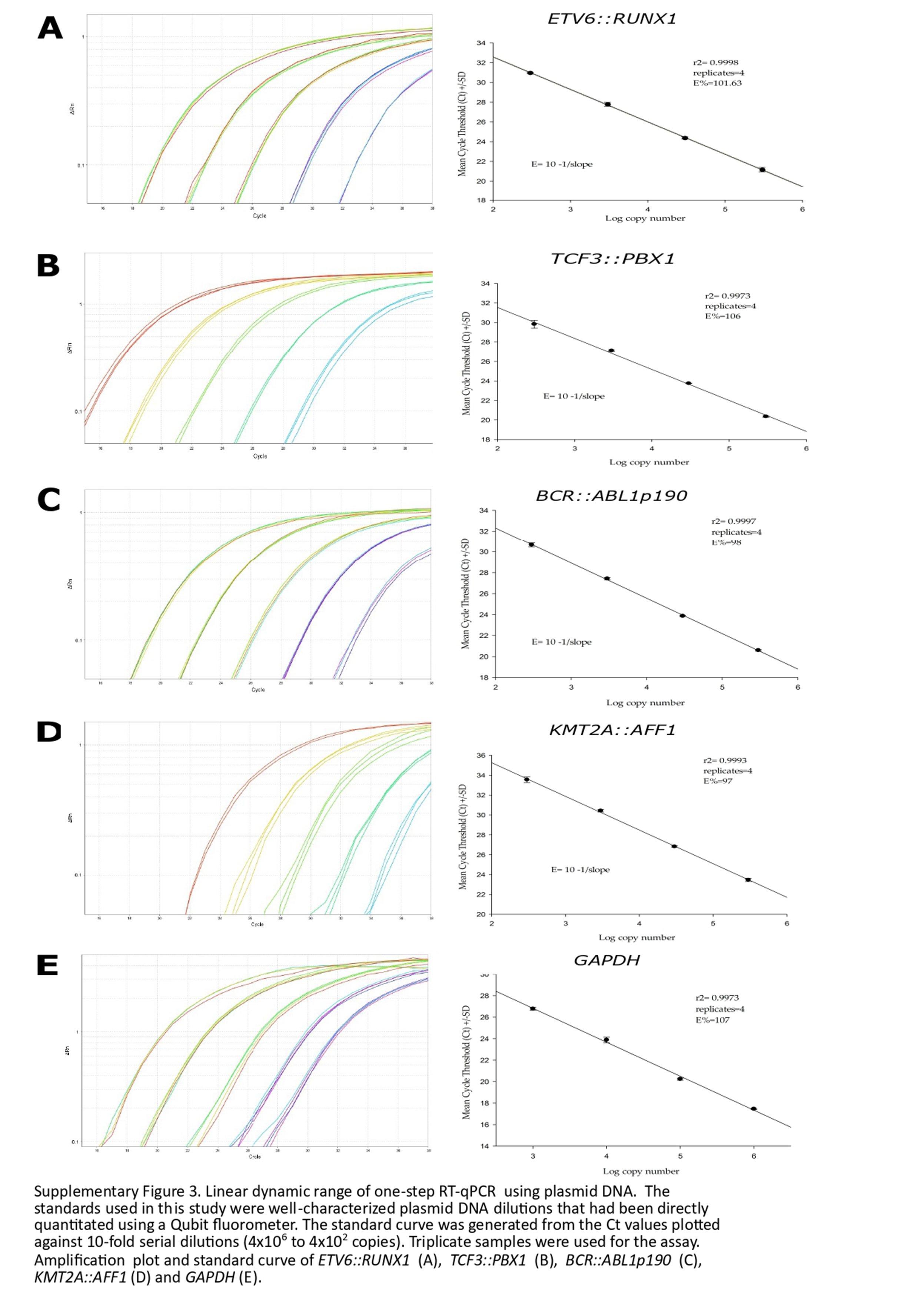

Supplement: Supplementary file 7 [file Image_3.JPEG]
